# Associations of Education With Overall Diet Quality Are Explained by Different Food Groups in Middle-aged and Old Japanese Women

**DOI:** 10.2188/jea.JE20200030

**Published:** 2021-04-05

**Authors:** Ayumi Hashimoto, Kentaro Murakami, Satomi Kobayashi, Hitomi Suga, Satoshi Sasaki, the Three-generation Study of Women on Diets and Health Study Group

**Affiliations:** Department of Social and Preventive Epidemiology, School of Public Health, The University of Tokyo, Tokyo, Japan

**Keywords:** education, diet quality, Japanese

## Abstract

**Background:**

The disparity of overall diet quality by personal educational attainment has been a public issue. However, it remains unknown which food groups contribute to the disparity. This cross-sectional study assesses which food groups explain associations between education and overall diet quality in Japanese women.

**Methods:**

A total of 3,788 middle-aged (mean age, 47.7 years) and 2,188 older women (mean age, 74.4 years), who lived in 47 prefectures in Japan, provided data on their education (low, middle, and high) and dietary intakes from a diet history questionnaire. A diet quality score (possible score 0–70) was calculated based on seven food components. Mean diet quality scores, with adjustment for lifestyle and neighborhood variables, were estimated by education using a general linear model, and Dunnett’s multiple comparison was conducted. Additionally, mean scores of each food component were estimated by education and compared using the same manner.

**Results:**

After adjustment for lifestyle and neighborhood variables, mean diet quality score of high or middle education was higher than low education for both generations. Middle-aged women with high and middle education had higher scores of ‘milk’, ‘snacks, confection, and beverages’, ‘fruits’, and ‘vegetable dishes’ than those with low education. Older women with high and middle education had higher scores of ‘sodium from seasonings’ and ‘fruits’ than those with low education.

**Conclusions:**

This study suggests that positive associations between education and diet quality are explained by different food groups in middle-aged and older Japanese women, which are independent of lifestyle and neighborhood variables.

## INTRODUCTION

The disparity of dietary intake by personal socioeconomic status (SES) has been a public issue.^1^^–^^4^ Especially, people with low education have consistently shown low level of overall diet quality, which may be partly due to lack of nutrition knowledge, cooking skills, or ability to use prevention messages.^1^^–^^4^ Considering that women influence their family’s diet, and their food preparation behaviors differ more greatly than men according to their educational level,^5^^–^^7^ it is important to understand mechanisms for associations between education and diet quality in women from a nutritional perspective.

Nevertheless, no study has investigated which food groups explain the associations between education and diet quality, although fruits and vegetables showed the most consistent association with education.^1^^–^^3^^,^^8^^,^^9^ Moreover, generation-specific associations in middle-aged and older women remain unknown. In previous studies, there is generational difference in time spent cooking^6^ and in food preference.^10^^,^^11^ An Australian study reported that education was more strongly associated with diet quality in adults than in the older people.^4^

In addition to individual lifestyle, such as smoking and physical activity, neighborhood contexts are potential mediators of associations between education and diet quality.^3^^,^^4^^,^^12^^–^^18^ Previous studies suggested that people with higher education tend to live in high SES areas^12^^,^^13^ with good access to supermarkets,^14^ or urban.^3^^,^^15^^,^^16^ Moreover, high SES areas mostly related to good diet quality.^3^^,^^4^^,^^12^^,^^15^^–^^18^ Therefore, adjustment for mediating effects by lifestyle and neighborhood variables are needed to investigate direct associations between education and diet quality.

The objective of this cross-sectional study is to assess which food groups explain associations between education and overall diet quality in middle-aged and older Japanese women, with adjustment for lifestyle and neighborhood variables.

## METHODS

### Survey design

The Three-Generation Study of Women on Diets and Health was conducted on dietetic students, their mothers, and grandmothers in northern and western Japan in 2011 and in eastern Japan in 2012.^19^^,^^20^ The present analysis used data from the mother’s and grandmother’s generation. Briefly, the survey was conducted in a total of 85 academic institutions with a nutrition department. The 7,016 students were requested to invite their mothers and grandmothers to join this study, and to distribute two questionnaires on dietary habits and lifestyle variables to those who had agreed to participate. In total, 4,044 mothers (response rate: 57.6%) and 2,332 grandmothers (response rate: 33.2%) answered both questionnaires. Mothers and grandmothers were considered middle-aged and older, respectively.

To analyze middle-aged women, participants aged 34–60 years were selected (*n* = 4,011). We excluded those living in eastern Japan who participated in the 2011 survey because their dietary habits and lifestyle would have been influenced by the Great East Japan Earthquake (*n* = 63). We also excluded those from the institution with the low response rate (*n* = 2), and those with missing information on the variables of interest (*n* = 158). To analyze older women, participants aged 61–94 years were selected (*n* = 2,320). We excluded those living in eastern Japan who participated in the 2011 survey (*n* = 47), and those from the institution with the low response rate (*n* = 1). We also excluded those with missing information on the variables of interest (*n* = 84). Consequently, the final sample sizes were 3,788 and 2,188 for middle-aged and older women, respectively.

This study was conducted according to the Declaration of Helsinki and all study procedure were approved by the Ethics Committee of the University of Tokyo Faculty of Medicine (no 3249). Written informed consent was obtained from each participant.

### Calculation of the diet quality score

Dietary habits during the preceding month were assessed using a validated comprehensive diet history questionnaire (DHQ)^21^^–^^23^ for middle-aged women and a validated brief-type diet history questionnaire (BDHQ)^21^^,^^22^ for older women. Briefly, the DHQ and the BDHQ cover the consumption frequency (and portion size in the DHQ) of selected foods commonly consumed in Japan, as well as general dietary behavior and usual cooking methods.^21^^–^^23^ Estimates of the intake of food (151 items in the DHQ and 58 items in the BDHQ) and energy were calculated using an ad hoc computer algorithm, which was based on the Standard Tables of Food Composition in Japan.^24^ A relative validity of the DHQ and BDHQ has been previously investigated among ninety-two women aged 31–69 years using a 16-d dietary record as reference, in terms of energy-adjusted estimates of food groups and nutrients.^21^^,^^22^ Using the information from the DHQ and the BDHQ, overall diet quality was estimated using a previously developed food-based diet quality score (eTable 1).^19^^,^^25^ This score is based on six components recommended in the Japanese Food Guide Spinning Top and sodium from seasonings (seven components in total). When intake was within the recommended range, a score of 10 was assigned to that component. Energy-adjusted values of dietary intake were calculated using the density method (amount per 7,531 kJ) to compare with the recommended values. For a participant who fell short of or exceeded the recommended value, the score was calculated proportionately between 0 and 10. The seven scores were then summed to provide the diet quality score, which ranged from 0 to 70. In the present population, a higher diet quality score was associated with favorable nutrient intake patterns, such as higher intakes of dietary fiber and micronutrients and lower intakes of saturated fat and sodium.^19^

### Education and lifestyle variables

All the variables were based on the participants’ self-reported information, except for the education of middle-aged women, which was obtained from their daughter’s or son’s questionnaires. Education was categorized as low, middle, and high. For middle-aged women, they corresponded to ≤12 years (ie, junior high school or high school), 13–15 years (ie, junior college or professional college), and ≥16 years (ie, university). For older women, they corresponded to ≤9 years (ie, junior high school), 10–12 years (ie, high school), and ≥13 years (ie, junior college, professional college, or university). Age at the time of the survey was calculated based on the birth date. Body mass index (BMI) was calculated as body weight (kg) divided by the square of body height (m). Living status (living alone or living with others), current marital status (married or widowed/divorced/never married), smoking status (current smoker, former smoker, or non-smoker), and prescription medicine use (yes or no) were also obtained. Physical activity was defined as the average total metabolic equivalent-hours score per day based on the frequency and duration of seven activities.^26^ Energy-adjusted diet cost (Japanese yen/4,184 KJ) was calculated in accordance with previous studies,^20^ by multiplying the consumption of each food item by food price using the 2012 Retail Price survey^27^ (141 items for DHQ, 57 items for BDHQ), supermarket websites (9 items for DHQ, 1 item for BDHQ), and fast-food restaurants (1 item for DHQ), and then summing these products. The employment status (housewife [not employed], part-time worker, or full-time worker) was obtained only for middle-aged women.

### Neighborhood variables

Neighborhood contexts were assessed using urban-rural classification, percentage of workers in the primary sector industry, areal deprivation index (ADI), number of food retailers, and region. In this study, neighborhood was defined as a municipality where a participant lived during the past month. A total of 1,147 municipalities from all 47 prefectures were covered (ie, 60.3% of the total municipalities in Japan). Urban-rural classification was defined in the 2010 population census,^28^ as central cities (Tokyo 23 districts, ordinance-designated cities, and other major cities), the suburbs (municipalities that not only surrounded a central city but also have a 1.5% or higher population commuting to the city), and rural areas (the other municipalities). The percentage of workers in the primary sector industry (agriculture, fishing, and forestry) was obtained from the 2010 population census.^28^ ADI, as a measure of neighborhood SES reported by Nakaya et al,^29^^,^^30^ consisted of weighted sums of deprivation-related census variables (proportion of old couple households, old single households, single-mother households, rent houses, sales and service workers, agricultural workers, blue collar workers, and unemployment rate) using the 2010 population census.^28^ The number of food retailers in a municipality was obtained from the economic census for business activity in 2012.^31^ Municipalities were categorized according to the region.

### Statistical analysis

All statistical analyses were performed for middle-aged and older women separately, using SAS statistical software package version 9.4 (SAS Institute, Cary, NC, USA). All reported *P* values are two-tailed, and *P* < 0.05 is considered statistically significant. First, basic characteristics of participants were described. Second, to investigate associations between education and the diet quality score, crude mean (model 1) and adjusted mean (model 2 and model 3) and their 95% confidence interval of diet quality score by education were estimated using a general linear model. Additionally, Dunnett’s multiple comparison was conducted with the use of low education as a reference category. In model 2, age and individual lifestyle variables were adjusted to investigate the associations between education and the diet quality score. Individual lifestyle variables were BMI, living status (only for older women because 99.2% of middle-aged women lived with others), current marital status, employment status (only for middle-aged women), smoking status, physical activity, prescription medicine use, and diet cost. In model 3, neighborhood variables (urban-rural classification, percentage of workers in the primary sector industry, ADI, number of food retailers, and region) were also adjusted for. In this study, the multilevel model was not used because of the small number of participants in a municipality (average: 3.6 for middle-aged women and 2.6 for older women) and because of the small municipality level variance in overall variance of the diet quality score (intraclass correlation coefficients for the null model: 0.008 for middle-aged women and 0.004 for older women). Third, the associations between education and the seven components of the diet quality score were estimated using the same manner.

## RESULTS

### Characteristics of participants

The characteristics of participants are shown in Table 1. The mean age of middle-aged and older women was 47.7 years and 74.4 years, respectively. In comparison to older women, middle-aged women had a lower mean BMI and a higher proportion of living with others, married, high education, current smoker, and non-user of prescription medicine. Additionally, middle-aged women had a higher score of total metabolic equivalent-hours, and spent less on diet than older women. The mean values of diet quality score and scores of ‘vegetable dishes’, ‘fish and meat dishes’, ‘fruits’, ‘snacks, confection, and beverages’, and ‘sodium from seasonings’ were higher in older women than middle-aged women. The intake of energy and total fat were on average higher in middle-aged women, while the intake of protein and carbohydrate were higher in older women. A higher proportion of middle-aged women lived in central cities than older women. Municipalities where middle-aged women lived had a lower percentage of workers in the primary sector industry and lower ADI, while no difference in the number of food retailers was found compared to older women.

**Table 1.** Characteristics of middle-aged and older Japanese women^a^

	Middle-aged (*n* = 3,788)		Older (*n* = 2,188)		*P*^e^
**Individual lifestyle variables**					
Age, years	47.7	3.9	74.4	5.2	<0.0001
Body mass index, kg/m^2^	22.0	3.1	22.8	3.2	<0.0001
Living status					
Alone	31	(0.8)	341	(15.6)	<0.0001
Live with others	3,757	(99.2)	1,847	(84.4)	
Marital status					
Married	3,468	(91.6)	1,332	(60.9)	<0.0001
Widowed/divorced/never married	320	(8.4)	856	(39.1)	
Education^b^					
Low	1,818	(48.0)	981	(44.8)	<0.0001
Middle	1,420	(37.5)	994	(45.4)	
High	550	(14.5)	213	(9.7)	
Employment status					
Housewife	742	(19.6)	—	—	—
Part-time worker	1,680	(44.4)	—	—	
Full-time worker	1,366	(36.1)	—	—	
Smoking status					
Current smoker	287	(7.6)	59	(2.7)	<0.0001
Former smoker	315	(8.3)	86	(3.9)	
Non-smoker	3,186	(84.1)	2,043	(93.4)	
Physical activity, total metabolic equivalent-hours score per day	40.7	5.6	39.0	6.6	<0.0001
Prescription medicine use					
Yes	958	(25.3)	1,684	(77.0)	<0.0001
No	2,830	(74.7)	504	(23.0)	
Diet cost, Japanese yen/4,184 kJ	568	108	643	124	<0.0001
Diet quality score^c^	42.9	8.1	50.5	8.0	<0.0001
Components of diet quality score^d^					
Grain dishes					
score	8.2	1.8	8.2	1.9	0.18
g/4,184 kJ	208.7	61.0	216.6	66.9	<0.0001
Vegetable dishes					
score	7.0	2.4	9.0	1.7	<0.0001
g/4,184 kJ	151.4	75.6	235.6	99.8	<0.0001
Fish and meat dishes					
score	9.8	0.8	9.9	0.5	<0.0001
g/4,184 kJ	113.8	40.1	153.3	50.4	<0.0001
Milk					
score	6.1	3.6	6.0	3.7	0.26
g/4,184 kJ	69.3	64.3	69.0	55.9	0.85
Fruits					
score	3.2	2.7	5.3	3.1	<0.0001
g/4,184 kJ	36.9	35.4	62.8	43.4	<0.0001
Snacks, confection, and beverages					
score	4.9	4.0	6.0	4.1	<0.0001
kJ/4,184 kJ	745.9	353.5	641.3	380.3	<0.0001
Sodium from seasonings					
score	3.7	3.6	6.1	2.9	<0.0001
mg/4,184 kJ	1,218.4	520.4	1,077.7	290.5	<0.0001
Energy, kJ/day	7,711.4	2,224.9	7,369.8	2,271.5	<0.0001
Nutrient intake					
Protein, % of energy	13.7	2.0	16.9	3.2	<0.0001
Total fat, % of energy	29.1	5.9	25.7	5.1	<0.0001
Carbohydrate, % of energy	54.3	7.1	55.9	7.4	<0.0001
**Neighborhood variables**					
Urban-rural classification					
Central cities	854	(22.5)	418	(19.1)	<0.0001
Suburbs	1,451	(38.3)	787	(36.0)	
Rural areas	1,483	(39.1)	983	(44.9)	
Percentage of workers in primary sector industry	4.5	5.8	5.1	6.2	0.0003
Areal deprivation index score	6.76	0.6	6.82	0.6	0.0004
Number of food retailers	325	281	335	294	0.18
Region					
Hokkaido and Tohoku	381	(10.1)	206	(9.4)	0.01
Kanto	1,074	(28.4)	544	(24.9)	
Hokuriku and Tokai	839	(22.1)	533	(24.4)	
Kinki	491	(13.0)	269	(12.3)	
Chugoku and Shikoku	514	(13.6)	353	(16.1)	
Kyushu	489	(12.9)	283	(12.9)	

^a^Data are shown as mean and standard deviation for continuous variables and number (percentage) of participants for categorical variables.

^b^For middle-aged women, low education is ≤12 years (junior high school or high school). Middle education is 13–15 years (junior college or professional college). High education is ≥16 years (university). For older women, low education is ≤9 years (junior high school). Middle education is 10–12 years (high school). High education is ≥13 years (junior college, professional college or university).

^c^Possible score ranging from 0 to 70.

^d^Possible score ranging from 0 to 10.

^e^Student’s *t*-test for continuous variables and pearson’s chi-square test for categorical variables.

### Associations of education with diet quality

As shown in Table 2 (for middle-aged women) and Table 3 (for older women), women with high and middle education had a higher total diet quality score than those with low education (model 1), even after adjustment for individual lifestyle variables (model 2), as well as individual lifestyle and neighborhood variables (model 3). However, the associations seemed to be due to different components of the diet quality score by generation. After adjustments for individual lifestyle and neighborhood variables (model 3), middle-aged women with high and middle education had higher scores of ‘milk’, ‘snacks, confection, and beverages’, ‘fruits’, and ‘vegetable dishes’ than those with low education. On the other hand, older women with high and middle education had higher scores of ‘sodium from seasonings’ and ‘fruits’ than those with low education. Although some lifestyle variables were associated with the diet quality score, no significant associations were found between neighborhood variables and diet quality except urban-rural classification (eTable 2).

**Table 2.** Associations of education with components of diet quality score in middle-aged women (*n* = 3,788)^a^

	Model 1^b^					Model 2^c^					Model 3^d^				

	Low	Middle		High		Low	Middle		High		Low	Middle		High	
	(*n* = 1,818)	(*n* = 1,420)		(*n* = 550)		(*n* = 1,818)	(*n* = 1,420)		(*n* = 550)		(*n* = 1,818)	(*n* = 1,420)		(*n* = 550)	

	mean (95% CI^e^)	mean (95% CI^e^)	*P*^f^	mean (95% CI^e^)	*P*^f^	mean (95% CI^e^)	mean (95% CI^e^)	*P*^f^	mean (95% CI^e^)	*P*^f^	mean (95% CI^e^)	mean (95% CI^e^)	*P*^f^	mean (95% CI^e^)	*P*^f^
Diet quality score^g^	41.7 (41.3–42.1)	43.4 (43.0–43.8)	<0.001	45.4 (44.7–46.0)	<0.001	42.0 (41.6–42.3)	43.3 (42.9–43.7)	<0.001	44.8 (44.1–45.5)	<0.001	42.0 (41.6–42.3)	43.3 (42.9–43.7)	<0.001	44.8 (44.1–45.5)	<0.001
Components of diet quality score^h^															
Grain dishes	8.2 (8.1–8.3)	8.2 (8.1–8.3)	0.97	8.1 (7.9–8.2)	0.27	8.1 (8.1–8.2)	8.2 (8.1–8.3)	0.22	8.2 (8.1–8.3)	0.48	8.1 (8.0–8.2)	8.2 (8.1–8.3)	0.11	8.2 (8.1–8.4)	0.22
Vegetable dishes	6.7 (6.6–6.8)	7.3 (7.2–7.4)	<0.001	7.5 (7.3–7.7)	<0.001	6.9 (6.8–7.0)	7.2 (7.1–7.3)	<0.001	7.2 (7.0–7.3)	0.01	6.9 (6.8–7.0)	7.2 (7.1–7.3)	<0.001	7.2 (7.0–7.4)	0.01
Fish and meat dishes	9.8 (9.8–9.8)	9.8 (9.8–9.9)	0.17	9.9 (9.9–10.0)	<0.001	9.8 (9.8–9.8)	9.8 (9.8–9.9)	0.75	9.9 (9.8–9.9)	0.07	9.8 (9.8–9.8)	9.8 (9.8–9.9)	0.84	9.9 (9.8–9.9)	0.12
Milk	5.6 (5.5–5.8)	6.3 (6.1–6.4)	<0.001	7.2 (6.9–7.5)	<0.001	5.7 (5.5–5.9)	6.2 (6.0–6.4)	<0.001	7.0 (6.7–7.3)	<0.001	5.7 (5.6–5.9)	6.2 (6.0–6.4)	<0.001	6.9 (6.6–7.2)	<0.001
Fruits	2.9 (2.8–3.0)	3.3 (3.1–3.4)	<0.001	3.8 (3.6–4.1)	<0.001	3.0 (2.9–3.2)	3.2 (3.1–3.4)	0.12	3.5 (3.3–3.7)	<0.001	3.0 (2.9–3.2)	3.2 (3.1–3.4)	0.09	3.5 (3.3–3.8)	<0.001
Snacks, confection, and beverages	4.6 (4.5–4.8)	5.0 (4.8–5.2)	0.01	5.4 (5.1–5.7)	<0.001	4.7 (4.5–4.9)	5.0 (4.8–5.2)	0.04	5.3 (5.0–5.6)	0.004	4.7 (4.5–4.8)	5.0 (4.8–5.2)	0.02	5.4 (5.0–5.7)	0.001
Sodium from seasonings	3.9 (3.7–4.0)	3.6 (3.4–3.7)	0.04	3.5 (3.2–3.8)	0.04	3.7 (3.6–3.9)	3.6 (3.4–3.8)	0.53	3.7 (3.4–4.0)	0.99	3.7 (3.6–3.9)	3.6 (3.4–3.8)	0.55	3.7 (3.4–4.0)	0.98

^a^Low education is ≤12 years (junior high school or high school). Middle education is 13–15 years (junior college or professional college). High education is ≥16 years (university).

^b^Model 1: crude model.

^c^Model 2: adjusted for age, body mass index, current marital status, employment status, smoking status, physical activity, prescription medicine use, and diet cost.

^d^Model 3: adjusted for variables in model 2, urban-rural classification, percentage of workers in primary sector industry, areal deprivation index score, number of food retailers, and region.

^e^Confidence interval.

^f^Dunnett’s multiple comparison was conducted (reference: low).

^g^Possible score ranging from 0 to 70.

^h^Possible score ranging from 0 to 10.

**Table 3.** Associations of education with components of diet quality score in older women (*n* = 2,188)^a^

	Model 1^b^					Model 2^c^					Model 3^d^				

	Low	Middle		High		Low	Middle		High		Low	Middle		High	
	(*n* = 981)	(*n* = 994)		(*n* = 213)		(*n* = 981)	(*n* = 994)		(*n* = 213)		(*n* = 981)	(*n* = 994)		(*n* = 213)	

	mean (95% CI^e^)	mean (95% CI^e^)	*P*^f^	mean (95% CI^e^)	*P*^f^	mean (95% CI^e^)	mean (95% CI^e^)	*P*^f^	mean (95% CI^e^)	*P*^f^	mean (95% CI^e^)	mean (95% CI^e^)	*P*^f^	mean (95% CI^e^)	*P*^f^
Diet quality score^g^	49.6 (49.1–50.1)	50.9 (50.4–51.4)	<0.001	52.4 (51.3–53.5)	<0.001	49.7 (49.2–50.2)	50.9 (50.4–51.3)	0.004	52.2 (51.2–53.3)	<0.001	49.8 (49.3–50.3)	50.8 (50.3–51.3)	0.013	52.2 (51.1–53.2)	<0.001
Components of diet quality score^h^															
Grain dishes	8.4 (8.3–8.5)	8.1 (8.0–8.3)	0.003	7.8 (7.6–8.1)	<0.001	8.3 (8.2–8.4)	8.2 (8.1–8.3)	0.75	8.2 (8.0–8.4)	0.86	8.3 (8.2–8.3)	8.2 (8.1–8.3)	0.72	8.2 (8.0–8.4)	0.96
Vegetable dishes	8.9 (8.8–9.0)	9.1 (9.0–9.2)	0.08	9.4 (9.2–9.7)	<0.001	9.0 (8.9–9.1)	9.0 (8.9–9.1)	0.98	9.2 (9.0–9.4)	0.20	9.0 (8.9–9.1)	9.0 (8.9–9.1)	0.98	9.2 (9.0–9.4)	0.17
Fish and meat dishes	9.9 (9.9–10.0)	9.9 (9.9–10.0)	0.55	10.0 (9.9–10.1)	0.48	10.0 (9.9–10.0)	9.9 (9.9–9.9)	0.24	10.0 (9.9–10.0)	0.98	10.0 (9.9–10.0)	9.9 (9.9–9.9)	0.19	10.0 (9.9–10.0)	0.99
Milk	5.6 (5.4–5.8)	6.3 (6.0–6.5)	<0.001	6.4 (5.9–6.8)	0.02	5.7 (5.5–5.9)	6.2 (6.0–6.4)	0.01	6.2 (5.7–6.7)	0.17	5.7 (5.5–5.9)	6.2 (6.0–6.4)	0.01	6.1 (5.6–6.6)	0.24
Fruits	4.8 (4.6–5.0)	5.5 (5.3–5.7)	<0.001	6.4 (6.0–6.8)	<0.001	4.9 (4.8–5.1)	5.4 (5.2–5.6)	<0.001	6.0 (5.6–6.4)	<0.001	5.0 (4.8–5.2)	5.4 (5.2–5.6)	0.004	6.0 (5.6–6.4)	<0.001
Snacks, confection, and beverages	6.0 (5.8–6.3)	6.0 (5.7–6.3)	0.98	5.7 (5.1–6.2)	0.43	6.1 (5.8–6.3)	6.0 (5.7–6.2)	0.77	5.6 (5.1–6.2)	0.28	6.1 (5.8–6.4)	5.9 (5.7–6.2)	0.61	5.7 (5.1–6.2)	0.29
Sodium from seasonings	5.9 (5.8–6.1)	6.1 (5.9–6.3)	0.42	6.7 (6.4–7.1)	<0.001	5.8 (5.6–6.0)	6.2 (6.0–6.3)	0.01	7.1 (6.7–7.4)	<0.001	5.8 (5.7–6.0)	6.1 (6.0–6.3)	0.02	7.0 (6.7–7.4)	<0.001

^a^Low education is ≤9 years (junior high school). Middle education is 10–12 years (high school). High education is ≥13 years (junior college, professional college or university).

^b^Model 1: crude model.

^c^Model 2: adjusted for age, body mass index, living status, current marital status, smoking status, physical activity, prescription medicine use, and diet cost.

^d^Model 3: adjusted for variables in model 2, urban-rural classification, percentage of workers in primary sector industry, areal deprivation index score, number of food retailers, and region.

^e^Confidence interval.

^f^Dunnett’s multiple comparison was conducted (reference: low).

^g^Possible score ranging from 0 to 70.

^h^Possible score ranging from 0 to 10.

## DISCUSSION

To our knowledge, this is the first study to investigate which food groups explain the associations between education and overall diet quality by generation. This study found that positive associations between education and diet quality were explained by different food groups in middle-aged and older Japanese women, namely by ‘milk’ and ‘sodium from seasonings’, which are independent of lifestyle and neighborhood variables.

In previous studies, education was positively associated with overall diet quality in both middle-aged and older women.^2^^–^^4^^,^^9^ This is consistent with this study. Interestingly, however, this study showed that associations between education and food components of the diet quality score differed by generation. The score difference between high and low education for middle-aged women was the largest for ‘milk’, followed by ‘snacks, confection, and beverages’, ‘fruits’, and ‘vegetable dishes’, while in older women, it was the largest for ‘sodium from seasonings’, followed by ‘fruits’. Although the exact reason is unknown, generation difference in time spent cooking^6^ and food preference^10^^,^^11^ may be related to the associations. In previous studies, old age was associated with longer time spent cooking,^6^ putting more value on nutrition and less value on cost^10^^,^^11^ and convenience.^10^ Therefore, the present study suggested that middle-aged women with high education tended to improve their diet quality by eating food that did not require long cooking, such as increasing their intake of ‘milk’, ‘fruits’, and ‘vegetable dishes’ (such as salad), and decreasing their intake of ‘snacks, confection, and beverages’. This is partly consistent with a study in France, which shows that middle-aged women with high education spent less time in meal preparation but prepared food from scratch (eg, use of raw or fresh ingredients) more frequently than those with low education.^7^ Conversely, older women with high education may improve their diet quality by cooking in a healthy manner, such as reducing their intake of ‘sodium from seasonings’, in addition to increasing their intake of ‘fruits’. Since the main source of salt intake in Japan was discretionary salt (57.1%),^32^ older women with high education may use seasoning more carefully than those with low education.

Moreover, the present study shows that associations between education and diet quality were independent of neighborhood variables, suggesting that the associations were not mediated by neighborhood contexts in this study. This may be partly because of limited association of food intakes with neighborhood SES^13^ or store availability in Japan.^18^ Actually, no significant associations were found in this study between neighborhood variables and diet quality except urban-rural classification.

The present findings might be useful for developing a public health policy to reduce disparity of diet quality by education in Japan. There may be a greater need to address the issue of ‘milk’, ‘snacks, confection, and beverages’, ‘fruits’, and ‘vegetable dishes’ for middle-aged women, while ‘sodium from seasonings’ and ‘fruits’ for older women.

The present study has several limitations. First, the participants were mothers and grandmothers of dietetic students, and not a random sample of Japanese middle-aged and older women. Nevertheless, the participants were comparable to the general middle-aged and older women in terms of education (low, high: 54%, 16% for the former, and 40%, 11% for the latter)^28^ and the diet quality score (mean 42.0; standard deviation, 8.9 for the former, and mean 47.1; standard deviation, 9.6 for the latter),^33^ but not in terms of living status (those living alone: 9%, 20%, respectively),^28^ or current marital status (those who are married: 72%, 54%, respectively).^28^ Second, neighborhood variables were measured using municipality level data because it was the smallest spatial unit available in this study. However, municipality may be too large to be used as a neighborhood influencing diet (average area size: 204.4 km^2^). Moreover, the data was not representative of each municipality and only included a small number of participants in each municipality (average: 3.6 for middle-aged women and 2.6 for older women). In addition, duration of residence was not taken into account for analysis because of a lack of information. Third, a self-report dietary assessment was conducted which is subject to measurement error particularly caused by the misreporting of food intake. To minimize measurement error, we assessed the dietary habits using a well-established assessment questionnaire (ie. DHQ and BDHQ) with reasonable validity^21^^–^^23^ as well as energy-adjusted dietary values for calculating the diet quality score. Additionally, exclusion of energy intake misreporters, evaluated by the ratio of energy intake to basal metabolic rate (the Goldberg cut-off),^19^^,^^20^^,^^34^ did not change most results (data not shown), which may support the robustness of the present finding. Fourth, because different dietary assessment questionnaires were used for middle-aged (DHQ) and older (BDHQ) women, it is not possible to directly compare differences in the diet quality score by education between generations. Nevertheless, the diet quality score was similarly associated with nutrient intakes in both generations.^19^ Fifth, we lacked data on personal or household income as a residual confounder. People with low income tended to choose low-cost foods, which are generally energy-dense and nutrient-poor.^1^ Since the association between income and diet quality may be partly mediated by diet cost,^1^ the dietary cost was adjusted in this study to minimize potential influence of income on the association of education with diet quality.

In conclusion, this study suggests that positive associations between education and diet quality are explained by different food groups in middle-aged and older Japanese women, which are independent of lifestyle and neighborhood variables. The present finding might be useful for developing a public health policy to reduce disparity of diet quality by education in Japan.

## APPENDIX A. SUPPLEMENTARY DATA

eTable 1. Scoring system of the diet quality score

eTable 2. Associations of individual lifestyle variables and neighborhood variables with diet quality score
