# Supplementary material for: Associations of Education With Overall Diet Quality Are Explained by Different Food Groups in Middle-aged and Old Japanese Women
Source: J Epidemiol. 2021 Apr 5;31(4):280–6. doi: 10.2188/jea.JE20200030 (PMC7940974; doi:10.2188/jea.JE20200030)
Supplement: Supplementary file 1 [file je-31-280-s001.pdf]

eTable 1. Scoring system of the diet quality score

Category[a]	Food groups	Food item		Diet quality score	
		Comprehensive diet history questionnaire (DHQ)[b]	Brief-type diet history questionnaire (BDHQ)[c]	Serving consumed[d]	Score given
Grain dishes	White rice	White rice (1 item)	White rice (1 item)	<4	10 × (serving consumed)/4
	Other grains	White rice mixed with barley; white rice with germ; half-milled rice; 70 %-milled rice; brown rice（5 items)	---[e]	≥4	10
	Noodles	Japanese noodles (buckwheat, instant noodles and Japanese wheat noodles); instant noodles; Chinese noodles; spaghetti and macaroni (4 items)	Buckwheat and instant noodles; Japanese wheat noodles; instant noodles and Chinese noodles; spaghetti and macaroni (4 items)		
	Bread	White bread; butter roll; croissant (3 items)	Breads (including white bread and Japanese bread with a sweet filling) (1 item)		
	Other grain products	Pizza; Japanese-style pancakes; cornflakes (3 items)	---[e]		
Vegetable dishes	Potatoes	French fries; potatoes; sweet potatoes, yams, and taro; konnyaku (i.e., devil's tongue jelly) (4 items)	Potatoes (all varieties) (1 item)	<5	10 × (serving consumed)/5
	Nuts	Peanuts; other nuts (2 items)	---[e]	≥5	10
	Total vegetable	Carrots; pumpkins; tomatoes; green peppers; broccoli; green leafy vegetables; salted pickled plums; cabbage; cucumbers; lettuce; Chinese cabbage; bean sprouts; radishes; onions; cauliflower; eggplants; burdock; lotus root; salted pickles (leafy vegetables); salted pickles (others); mushrooms; wakame and hijiki seaweed; laver (i.e., dried, edible seaweed); vegetable juice; tomato juice (25 items)	Salted green and yellow vegetable pickles; other salted vegetables (excluding salted pickled plums); raw vegetables used in salad (e.g., cabbage and lettuce); green leafy vegetables including broccoli; cabbage and Chinese cabbage; carrots and pumpkins; radishes and turnips; other root vegetables (e.g., onions, burdock, and lotus root); tomatoes, tomato ketchup, boiled tomato, and stewed tomato; mushrooms (all varieties); seaweeds (all varieties); vegetable juice (12 items)		

Fish and meat dishes	Meat	Ground beef and pork; chicken; pork; beef; liver; ham and sausages; bacon (7 items)	Chicken (including ground chicken); pork and beef (including ground and beef); ham, sausages, and bacon; liver (4 items)	<3	10 × (serving consumed)/3
	Egg	Eggs (1 item)	Eggs (1 item)	≥3	10
	Pulses	Tofu (i.e., soybean curd); tofu products; natto (i.e., fermented soybeans); boiled beans; soy milk (5 items)	Tofu (i.e., soybean curd) and tofu products; natto (i.e., fermented soybeans) (2 item)		
	Fish and selfish	Dried fish; small fish with bones; canned tuna; eel; white meat fish; red meat fish; oily fish; ground fish meat products; shrimp and crab; squid and octopus; oysters; other shellfish; fish eggs; boiled fish and shellfish in soy sauce; salted fish intestines (15 items)	Squid, octopus, shrimp, and clam; small fish with bones; canned tuna; dried fish and salted fish (including salted mackerel, salted salmon, and dried horse mackerel); oily fish (including sardines, mackerel, saury, amberjack, herring, eel, and fatty tuna); non-oily fish (including salmon, trout, white meat fish, freshwater fish, and bonito) (6 items)		
Milk	Dairy products	Sweetened yogurt; non-sweetened yogurt; moderately sweetened yogurt; cheese; cottage cheese; low-fat milk; full-fat milk; cream or creamer added to coffee (8 items)	Low-fat milk and yogurt; full-fat milk and yogurt (2 items)	<2	10 × (serving consumed)/2
				≥2	10
Fruits	Fruit	Raisins; canned fruits; oranges; bananas; apples; strawberries; grapes; peaches; pears; persimmons; kiwi fruits; melons; watermelons; fruit juice (14 items)	Citrus fruit; strawberries, persimmons, and kiwi fruits; other fruit (3 items)	<2	10 × (serving consumed)/2
				≥2	10
Snacks, confection, and beverages	Alcoholic	Beer; Sake; Shochu; Shochu mixed with water or a carbonated beverage;	Beer; Sake; Shochu; whiskey; wine; (5 items)	≤837 kJ	10
	beverages	whiskey; wine; (6 items)		>837 kJ	10 - 10 × [(energy consumed in kJ) - 837]/837[f]
	Sugar and confectioneries	Jam and marmalade; sugar for coffee and black tea; Japanese bread with a sweet filling; pancakes; potato chips; rice crackers; snacks made from wheat flour; Japanese sweets with azuki beans; Japanese sweets without azuki beans; cakes; cookies and biscuits; chocolates; candies, caramels, and chewing gum; jellies; doughnuts; ice cream (premium); ice cream (unspecified varieties); ice cream (regular) (18 items)	Cakes, cookies, and biscuits; Japanese sweets; rice crackers, rice cakes, and Japanese-style pancakes; ice cream; sugar for coffee and black tea (5 items)		

	Soft drinks	Fruit juice excluding 100 % juice; cocoa; lactic acid bacteria beverages; sugar-sweetened soft drinks; nutritional supplement drinks (5 items)	Cola and sugar-sweetened soft drinks (1 item)		
Sodium from seasonings	Seasonings	Ketchup; fat-free salad dressing; table salt; salt used during cooking; soy sauce; curry and roux in stew; miso as seasoning (7 items)	Table salt; soy sauce (2 items)	For middle-aged women	
				≤1217 mg[g]	10
				>1217 mg[g]	10-10×((sodium consumed in mg)-1217[g])/ 1217[f]
				For older women	
				≤1389 mg[g]	10
				>1389 mg[g]	10-10×((sodium consumed in mg)-1389[g])/ 1389[f]

[a]By definition, one serving of grain dishes contained 40 g carbohydrate; one serving of vegetable dishes was 70 g; one serving of fish and meat dishes contained 6 g protein; one serving of milk contained 100 mg calcium; and one serving of fruits was 100 g.

[b]Used for middle-aged women. The following food groups were not categorized and thus not used in the calculation described here: tea and coffee (3 items), sugar-free soft drinks (1 item), fats and oils (5 items), sugar used during cooking (1 item), soups (5 items), nutritional supplement bars (1 item), artificial sweeteners (1 item), and drinking water (1 item).

[c]Used for older women. The following food groups were not categorized and thus not used in the calculation described here: tea and coffee (3 items), fats and oils (2 items), sugar used during cooking (1 item), and soups (2 items).

[d]Per 7531 kJ of energy.

[e]Not available in BDHQ.

[f]When the calculation produced a negative score because of excess servings, energy, or sodium, the score was converted to 0.

[g]The values were based on the 10th percentile of energy-adjusted sodium intake from seasonings (per 7531 kJ) in middle-aged women (1217 mg of sodium) and in older women (1389 mg of sodium).

eTable 2. Associations of individual lifestyle variables and neighborhood variables with diet quality score

	Middle-aged (n=3,788)				Older (n=2,188)			
	β	95% CI[a]		*P*	β	95% CI[a]		*P*
Intercept	28.7	23.3	34.1	<0.0001	46.4	38.2	54.6	<0.0001
**Individual lifestyle variables**								
Age (per 1 year)	0.2	0.2	0.3	<0.0001	-0.05	-0.1	0.02	0.18
Body mass index (per 1 kg/m^2)	-0.03	-0.1	0.1	0.47	-0.1	-0.2	0.05	0.27
Living status (reference: alone)								
Live with others	-	-	-	-	-3.2	-4.2	-2.1	<0.0001
Marital status (reference: married)								
Widowed/divorced/never married	0.01	-1.0	1.0	0.99	-0.3	-1.1	0.5	0.48
Employment status (reference: housewife)								
Part-time worker	-1.3	-2.0	-0.6	<0.001	-	-	-	-
Full-time worker	-1.1	-1.9	-0.4	0.003	-	-	-	-
Smoking status (reference: current smoker)								
Former smoker	2.4	1.1	3.7	<0.001	3.4	0.6	6.2	0.02
Non-smoker	4.2	3.2	5.3	<0.0001	5.4	3.1	7.7	<0.0001
Physical activity (per 1 total metabolic equivalent-hours score per day)	0.03	-0.01	0.1	0.16	0.1	0.01	0.1	0.03
Prescription medicine use (reference: no)								
Yes	-0.4	-0.9	0.2	0.22	1.7	0.9	2.6	<.0001
Diet cost (per 1000 Japanese yen/4184 kJ)	-0.6	-3.1	1.9	0.65	1.9	-1.0	4.8	0.21
**Neighborhood variables**								
Urban-rural classification (reference: central cities)								
Suburbs	1.0	0.3	1.7	0.01	-0.2	-1.1	0.8	0.73
Rural areas	0.8	0.1	1.6	0.03	-1.1	-2.0	-0.1	0.04
Workers in primary sector industry (per 1% of workers)	0.02	-0.03	0.1	0.41	-0.03	-0.1	0.04	0.42
Areal deprivation index score (per 1 score)	-0.2	-0.7	0.3	0.36	0.2	-0.5	0.8	0.60
Number of food retailers (per 1000 food retailers)	-0.2	-1.2	0.8	0.67	0.2	-1.1	1.5	0.79
Region (reference: Kanto)								
Hokkaido and Tohoku	0.5	-0.5	1.5	0.31	0.7	-0.6	2.0	0.29
Hokuriku and Tokai	-0.4	-1.2	0.3	0.23	0.5	-0.5	1.6	0.29
Kinki	-0.6	-1.6	0.3	0.17	1.0	-0.3	2.2	0.13
Chugoku and Shikoku	-0.7	-1.6	0.1	0.09	1.3	0.2	2.4	0.02
Kyushu	-0.3	-1.2	0.7	0.56	0.9	-0.3	2.2	0.14

[a]Robust 95% confidence intervals (CIs) were obtained from generalized estimating equations.
